# An Automated Conversational Agent Self-Help Program: Randomized Controlled Trial

**DOI:** 10.2196/53829

**Published:** 2024-12-06

**Authors:** Heather M Foran, Christian Kubb, Janina Mueller, Spencer Poff, Megan Ung, Margaret Li, Eric Michael Smith, Akinniyi Akinyemi, Melanie Kambadur, Franziska Waller, Mario Graf, Y-Lan Boureau

**Affiliations:** 1 Department of Health Psychology University of Klagenfurt Klagenfurt Austria; 2 Meta AI Fundamental Research Menlo Park, CA United States

**Keywords:** well-being, chatbot, randomized controlled trial, prevention, flourishing

## Abstract

**Background:**

Health promotion and growth-based interventions can effectively improve individual well-being; however, significant gaps in access and utilization still exist.

**Objective:**

This study aims to develop and test the effectiveness and implementation of a new, widely targeted conversational agent prevention program (Zenny) designed to enhance well-being.

**Methods:**

A total of 1345 individuals in the United States were recruited online and randomly assigned to either (1) a self-help program intervention delivered via an automated conversational agent on WhatsApp or (2) an active control group that had access to evidence-based wellness resources available online. The primary outcomes were well-being (measured using the 5-item World Health Organization Well-being Scale), psychosocial flourishing (assessed with the Flourishing Scale), and positive psychological health (evaluated with the Mental Health Continuum-Short Form). Outcome measures were collected at baseline and again 1 month postassessment. All analyses were conducted using an intention-to-treat approach.

**Results:**

Both groups showed significant improvements in well-being (self-help program intervention group effect size: Cohen d=0.26, *P*<.001; active control group effect size: d=0.24, *P*<.001), psychosocial flourishing (intervention: d=0.19, *P*<.001; active control: d=0.18, *P*<.001), and positive psychological health (intervention: d=0.17, *P*=.001; active control: d=0.24, *P*<.001) at postassessment. However, there were no significant differences in effectiveness between the 2 groups (P ranged from .56 to .92). As hypothesized a priori, a greater number of days spent actively engaging with the conversational agent was associated with larger improvements in well-being at postassessment among participants in the intervention group (β=.109, *P*=.04).

**Conclusions:**

The findings from this study suggest that the free conversational agent wellness self-help program was as effective as evidence-based web resources. Further research should explore strategies to increase participant engagement over time, as only a portion of participants were actively involved, and higher engagement was linked to greater improvements in well-being. Long-term follow-up studies are also necessary to assess whether these effects remain stable over time.

**Trial Registration:**

ClinicalTrials.gov NCT06208566; https://clinicaltrials.gov/ct2/show/NCT06208566; OSF Registries osf.io/ahe2r; https://doi.org/10.17605/osf.io/ahe2r

## Introduction

### Background

Subjective well-being is relevant not only to psychological health but also to social relationships, work productivity, job satisfaction, and other health domains [1,2]. Fortunately, a large body of scientific knowledge exists on methods for promoting subjective well-being [3]. Health promotion and growth-based interventions can effectively enhance individuals’ life satisfaction [4].

Despite the promise of these interventions, they are rarely disseminated or implemented at the population level. This limited reach is due to inadequacies within health care systems, economic barriers, perceived stigma, and low accessibility of evidence-based health promotion tools [5,6]. A positive, growth-based approach focused on building self-help skills, rather than addressing problems, may help reduce stigma at the community level. Skills essential for well-being—such as emotion regulation, problem-solving, and other cognitive behavioral techniques [7,8]—can be taught early and may help prevent mental health issues later in life.

### Internet-Based or App-Based Programs

Several internet-based interventions for subjective well-being are supported by evidence from randomized controlled trials [9-11]. Delivering prevention programs online may help reduce barriers such as appointment scheduling, time commitments, travel, and intervention costs [12]. Additionally, offering these interventions in a private, self-help format may lower the perceived stigma associated with psychological self-improvement. Previous research has found that chatbot technology can be a valuable tool for reducing the stigma associated with mental illness and improving access to mental health resources [13]. Another common reason individuals avoid formal services is a preference to manage their problems independently [14]. Self-help programs offered in a self-paced format may foster a greater sense of personal improvement.

### The Next Step for Prevention

Despite their potential, many online interventions remain static and lack personalization to address individual needs, resulting in low dissemination and high dropout rates in trials [15,16]. Additionally, the limited availability of free online interventions reduces their public health impact. Automated conversational agents could provide a more tailored approach to enhance engagement and accessibility. A growing body of research explores conversational agent–based interventions in mental health, utilizing diverse study designs (eg, pilot feasibility studies, randomized controlled trials, quasi-experimental studies), technologies (eg, conversational agents accessed through apps, online platforms, offline computer programs, virtual reality headsets), intervention approaches (eg, cognitive behavioral therapy, psychoeducation), and mental health outcomes (eg, depression, anxiety, psychological distress, well-being) [17-20].

One of the earliest studies in this area tested a conversational agent named Woebot in a small sample of students (N=70) with elevated depressive and anxiety symptoms, yielding promising results [21]. Participants reported high satisfaction and showed improvements in depressive symptoms. However, the relevance of large-scale prevention remains uncertain, as the sample was selected based on elevated symptoms and consisted of a convenience sample of university students. In recent years, several other conversational agents targeting well-being or related constructs have been introduced, such as Shim [22], EMMA [23], Vivibot [24], and the 21-Day Stress Detox [25]. Similar to Woebot, their relevance and effectiveness for universal prevention remain unclear, often due to small sample sizes—ranging from 28 [22] to 64 participants [25]—or the lack of control groups [26].

While there are promising conversational agent–based interventions designed to promote mental health and well-being, fewer studies assess their impact on well-being outcomes compared with other mental health outcomes [20]. Furthermore, previous reviews highlight a lack of robust experimental designs, particularly randomized controlled trials using nonclinical samples, to demonstrate the efficacy of these conversational agent interventions for mental health and well-being [17,27]. Additionally, a significant number of studies examining conversational agents have used various psychological interventions without a solid theoretical foundation [17] or lack standardized measurement outcomes [19]. Finally, fostering greater interdisciplinary collaboration between computer science and mental health disciplines may help facilitate progress in this area [18].

In this study, we aimed to develop and test the effectiveness of a new conversational agent prevention program designed to enhance well-being and promote psychological growth. This project focuses not only on assessing the program’s effectiveness on well-being compared with an online active control condition in a randomized controlled trial but also on increasing accessibility for individuals who may benefit from such an intervention. Thus, the study uses brief, low-burden assessments instead of a full battery typical of psychological studies to enhance acceptability, enrollment rates, and retention rates. We used the RE-AIM (Reach, Effectiveness, Adoption, Implementation, and Maintenance) implementation science model [28] as a framework for this study. By adapting the model for our digital intervention, we address the following components in this paper: recruitment strategies, response rates, and enrollment rates (reach); changes in outcomes (effectiveness); and user satisfaction and participation/engagement (implementation).

### Hypotheses

- H1: The intervention group will show significant improvements in well-being and psychological flourishing at the 1-month postassessment compared with the control group.
- H2: The effectiveness of the intervention will be similar across gender and age after controlling for participation/engagement and user satisfaction.
- H3: The intervention group will have higher retention rates and report greater active participation/engagement than the control group.
- H4: Within the intervention group, higher user satisfaction and greater active participation/engagement will be associated with improved well-being.
- H5: Within the intervention group, higher user satisfaction while using the conversational agent will lead to increased participation/engagement over time.

## Methods

### Ethics Approval

The study was approved by the Western Institutional Review Board (WCG) in the United States (approval number 1289179). All hypotheses and planned analyses for this study were registered on the Open Science Framework platform [29] and in ClinicalTrials.gov. A priori power analysis is detailed in our study preregistration, indicating that the minimal sample size required to detect small effects in overall analyses was 216, with α<.05 and 95% power. The minimal sample size required for this analysis to detect small effects is 328, based on 2 assessment time points (α<.05 and 95% power). Copies of the informed consent forms can be requested by contacting the first author (HMF).

### Participants

Participants were recruited through advertising campaigns on Facebook (Meta Platforms, Inc.) and Instagram (Meta Platforms, Inc.) from July 2022 to April 2023. Eligibility criteria included being at least 18 years of age, English speaking, and residing in the United States with online access. After providing online informed consent, participants completed a brief questionnaire to assess their eligibility, demographics, and well-being. Participants were randomly assigned to either the conversational agent intervention or a selection of 3 evidence-based wellness resources linked from the study website. No financial compensation was provided for participation. Safety and security procedures were implemented, and participants were given referrals throughout the study. The procedures for data protection were detailed in the informed consent forms.

The registration process was initiated 2054 times, which included an eligibility check, informed consent, and the preassessment survey, followed by randomization into one of the groups. A total of 1349 participants provided consent and completed the preassessment. Four participants were lost due to a technical error during the preassessment, resulting in a sample of 1345 participants who completed the preassessment and were available for intent-to-treat analyses. The study flowchart is presented in the Results section.

### Study Design

This study uses a 2 (group: intervention and active control) × 2 (time: preassessment and 1-month postassessment) design. An overview of the full study is available in the clinical trials registry (NCT06208566). The intervention consisted of a conversational agent universal prevention program designed to enhance well-being. The control group received referrals to a selection of web-based, empirically supported mental health information. Completion of the interventions was expected to take approximately 1 month, depending on the individual level of participation and engagement.

### Randomization

Participants were randomized using a 1:1 allocation ratio with a block size of 10 from June 4, 2022, to July 29, 2022. A scientist not involved in the intervention conducted the randomization using a code developed specifically for this study. Participants were automatically assigned to 1 group after providing informed consent and completing the preassessment. After reaching the sample size necessary to detect effects in effectiveness based on a priori power analysis, and due to difficulties in recruitment, the randomization ratio was adjusted to 3:1 in favor of the intervention group on July 29, 2022. This change allowed for the recruitment of a larger sample for within-condition analyses (H4 and H5) while maintaining sufficient power to detect effects in the between-condition analyses. Participants were not blinded to the intervention to which they were assigned; however, they were aware of whether they were assigned to the intervention of interest or the comparator.

### Intervention Conditions

#### Intervention Condition: Conversational Agent Zenny

The intervention group consists of a newly developed automated conversational agent self-care program, which includes 40 separate modules based on empirically supported techniques. The modules cover the following core sections: cognitive behavioral skills, interpersonal relationships, positive psychological growth, relaxation, goal setting, and emotional regulation skills. The modules emphasize cognitive behavioral skills aimed at reducing emotional distress, enhancing behavioral activation and healthy behaviors, and improving problem-solving skills. The program’s goal is to introduce psychological concepts that have been shown to effectively manage emotions and improve personal efficiency to a broader public audience. Additionally, it aims to help individuals connect with widely available resources related to well-being. Individuals interested in self-help interventions often struggle to find appropriate resources, and the interventions they do find (eg, apps) may lack scientific validity. Providing personalized recommendations for resources tailored to an individual’s needs can help reduce this barrier to care. The intervention was delivered through a decision tree–based conversational agent on WhatsApp Messenger (Meta Platforms, Inc.), with participants contacted daily for 1 month. Participants received messages to encourage regular engagement with new modules or prompts to revisit previously completed ones. The conversational agent allowed participants to choose which topics to discuss and which branches of those topics to explore in greater depth. It also provided reminders about the content that participants themselves selected. Before the study, revisions were made based on detailed feedback from various team members and experts. After completing the registration, participants received an access code to connect with the conversational agent named *Zenny* at no cost.

#### Active Control Condition: Web-Based Wellness Resources

The control group received referrals to a menu of freely available evidence-based wellness resources linked from the study website, including *Your Healthiest Self: Wellness Toolkits* [30], *Doing What Matters in Times of Stress* [31], and *How Right Now* [32]. Participants in this group could self-select relevant modules aimed at improving mood, reducing stress and anxiety, addressing relationship issues, and exploring other wellness topics. These programs required only access to the websites and could be completed in a self-help format at the participants’ own pace. The resources were selected through a review of freely available online materials covering similar topics to those addressed by *Zenny*, with the aim of promoting well-being. They were chosen based on the criteria of being evidence-informed and sourced from credible organizations. Instead of offering a single resource option, three were selected to better reflect real-world scenarios, where individuals can choose from various online options.

### Measures

Assessments were conducted online through LimeSurvey (LimeSurvey GmbH) before randomization (preassessment) and again after 1 month for the postassessment (mean 39.80 days, SD 15.63 days). Participants received 3 email reminders to encourage their participation in the postassessment. Additionally, implementation data were collected exclusively for the conversational agent condition to further understand the impact of participation and engagement on outcomes. Demographic characteristics, including age and gender, were assessed for all participants during the preassessment. Qualitative data were collected at the end of the postassessment to gather feedback on participants’ study experiences. The following outcomes were included based on the RE-AIM framework.

### Measures of Reach

#### Recruitment Strategies

During the preassessment, participants were asked how they learned about the study (eg, through a group, advertisement, friend referral, or website). This information was collected to identify which recruitment strategies yielded the highest number of participants.

#### Enrollment Rate

This is calculated by dividing the number of participants who completed the preassessment by the total number of individuals who accessed the survey page during the recruitment period. The response rate, as defined in the study protocol, is the number of eligible participants who agreed to participate divided by the total number of visitors to the study page. However, to respect privacy, we did not collect data on the number of visitors and cannot identify unique visitors to report this information.

#### Retention Rate

This is defined as the number of participants who completed the 1-month postassessment.

### Primary Outcomes Measures of Effectiveness

#### Well-Being

The 5-item World Health Organization Well-Being Scale (WHO-5) [33] is a widely used self-reported measure of current subjective well-being. This measure is well-validated and commonly utilized in a web-based format. Participants are asked to rate their well-being over the past 2 weeks using a Likert scale from 0 to 5, with responses ranging from “0=At no time” to “5=All of the time” (eg, “My daily life has been filled with things that interest me”). Raw scores on the WHO-5 range from 0 to 25 and are then multiplied by 4, resulting in a final score range of 0 to 100, where higher scores indicate higher well-being. A systematic review has demonstrated that the measure possesses adequate validity for assessing well-being over time in nonclinical samples [34]. In this study, the internal consistency of the WHO-5 was high, with a Cronbach α of 0.89 and 0.91 at pre- and postassessment.

#### Psychosocial Flourishing

The Flourishing Scale (FS) developed by Diener et al [35] is an 8-item measure of positive human functioning. It assesses aspects of flourishing, including positive relationships, feelings of competence, a sense of meaning and purpose in life, and engagement in daily activities. Participants rate each statement on a 7-point scale, ranging from 1 (strongly disagree) to 7 (strongly agree). Total scores can range from 8 to 56, with higher scores indicating greater well-being. In this study, the internal consistency of the FS was excellent, with a Cronbach α of 0.91 and 0.93 at pre- and postassessment.

#### Positive Psychological Health

The Mental Health Continuum-Short Form (MHC-SF) by Keyes [36] assesses positive psychological health through 14 items organized into 3 subscales: Emotional Well-Being, Social Well-Being, and Psychological Well-Being. Previous research indicates that the Psychological Well-Being subscale is highly correlated with the overall well-being factor, often yielding similar scores to the total scale [37]. Therefore, this study utilized the 6-item Psychological Well-Being subscale (PWB), which includes items focused on self-acceptance, environmental mastery, positive relationships with others, personal growth, autonomy, and purpose in life. Respondents rate the frequency of each feeling experienced in the past month using a 6-point Likert scale, where 1 represents “never” and 6 indicates “every day.” In our sample, the Psychological Well-Being subscale of the MHC-SF demonstrated good internal consistency, with a Cronbach α of 0.86 and 0.89 at pre- and postassessment.

### Postassessment Measures of Implementation

#### User Satisfaction

This was assessed during the postassessment for both conditions using 4 items. Satisfaction with the program was evaluated across dimensions of satisfaction, usefulness, relevance, and helpfulness. Participants rated these aspects on a 4-point Likert scale, ranging from 1 (strongly disagree) to 4 (strongly agree). The item regarding usefulness was rated on a 5-point Likert scale. A sum score was calculated by aggregating all individual item scores, resulting in a range from 4 to 17, where higher scores indicate greater satisfaction with the content. The scale demonstrated excellent internal consistency within our sample (Cronbach α=0.92). The user satisfaction scale was modified to include only 4 items instead of 6 to reduce redundancy and shorten the survey length. This modified version was submitted for ethical review and included in the study protocol before its initiation, although it deviated from the original version registered on the Open Science Framework [29].

#### Participation/Engagement

This was assessed based on the reported frequency of using the intervention or control condition resources at the postassessment (2 items, *r*=0.79). In the first item, participants rated how often they participated in the last month on a 6-point Likert scale, ranging from “1=not at all” to “6=daily”. In the second item, participants rated their perceived level of activity on a 4-point Likert scale, ranging from “1=not at all active” to “4=very active.” The scores for both items were combined into a sum score, with higher scores indicating greater participation and engagement.

### Intervention Conversational Agent Measures of Implementation

For the intervention within-condition analyses, the implementation measures listed in Textbox 1 were assessed.

**Implementation measures for the within-condition analyses.**

1. Module rating of user satisfaction
  - This was assessed within the intervention group following each completed module. Users rated their satisfaction on a scale from 1 (not at all) to 5 (extremely). An average satisfaction rating across modules over the 30-day period was computed. Additionally, daily module ratings were utilized for within-condition analyses (H5).
2. Days chatted with the bot
  - Engagement with the conversational agent was quantified by counting the number of days each user interacted with the bot over the past 30 days, ranging from 0 to 30 days.
3. Total messages
  - In the intervention group, the total number of messages sent by users to the conversational agent was measured as an objective indicator of engagement. This metric includes any instance where the user initiated interaction with the conversational agent or responded to its messages within the past 30 days, with counts ranging from 0 to 30 days.
4. Modules started
  - This was the total number of modules that were initiated by the participant within the first 30 days, whether they were completed or not (ie, the sum of finished and unfinished modules).
5. Modules completed
  - This is the number of modules that were completed within the first 30 days (ie, finished modules).
6. Modules started not completed
  - This is the number of modules that were started but not completed within the first 30 days (ie, unfinished modules).

### Data Analytical Strategy

Data from all participants who were randomly assigned at preassessment were analyzed using an intention-to-treat approach. Before analysis, data underwent thorough quality assurance checks to ensure accuracy and reliability. As a result of the required response format during the preassessment, no missing data were identified for the outcome measures. A small amount of data was missing for age (22/1345, 1.64%) due to data entry errors (involving 3-digit ages) and for gender (9/1345, 0.67%) because identifying gender was not a mandatory response for participation. Before testing the key hypotheses, baseline differences between the intervention and control groups were assessed using chi-square tests and *t* tests (2-tailed and unpaired), as appropriate.

For analyses of effectiveness (H1), separate analyses were conducted to assess both within-group and between-group effects, evaluating the magnitude of change in the outcome variables at postassessment (WHO-5, PWB, and FS). Full information maximum likelihood estimation with robust statistics was applied using Mplus 8.6 [38]. Within-group effects were assessed using a Wald chi-square test to evaluate changes in outcome variables at postassessment for both the intervention and control groups. Between-group effects were estimated through an analysis of changes approach, with preassessment data adjusted [39]. Additionally, to investigate whether between-group effects varied by gender and age, interaction effects were included in the models for age × group and gender × group (H2), while controlling for participation/engagement and user satisfaction. For our third hypothesis (H3) concerning differences between conditions, we compared retention rates across groups using chi-square tests. Additionally, self-reported participation and engagement at postassessment were analyzed using linear regression models, consistent with the previously outlined analytical approaches.

For the fourth hypothesis (H4), we conducted within-group analyses of implementation outcomes specifically for the intervention group. Path analyses were performed to investigate whether greater changes in well-being over time, as measured by change scores in the WHO-5, PWB, and FS, could be predicted by engagement metrics. These metrics included module ratings, the number of days users interacted with the bot, and the total number of messages sent to the conversational agent. Additionally, self-reported user satisfaction, participation, and engagement measured at postassessment were included in the analysis. The models also controlled for age, gender, and preassessment levels. To evaluate whether higher user satisfaction, as indicated by module ratings, predicted subsequent increases in participation and engagement over the first 30 days after enrollment, we developed a multilevel model. This model accounted for elapsed time since enrollment and was implemented using Mplus statistical software, applying 2-level maximum likelihood estimation with robust statistics. Associations between average user satisfaction, as indicated by module ratings, and average participation/engagement over the 30 days are also reported. A significance level of *P*<.05 was utilized to determine statistical significance across all analyses. Additionally, Cohen *d* was presented as a measure of effect size, providing a standardized metric for evaluating the magnitude of group differences in the study. Larger Cohen *d* values indicate greater effect sizes [40].

## Results

### Recruitment Strategies and Enrollment Rate

**Figure 1:**
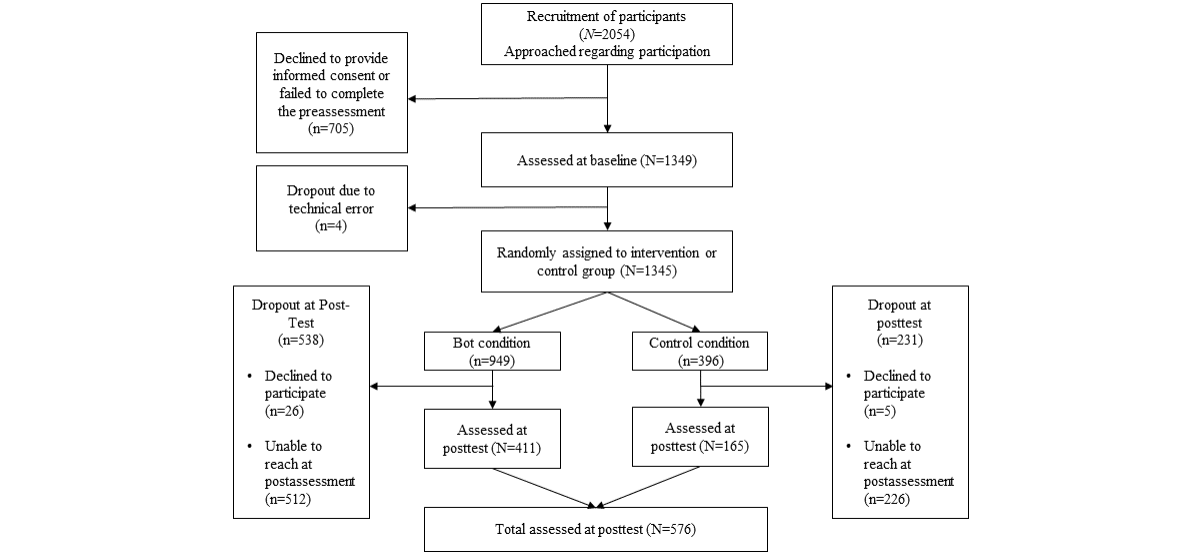
Study Sample Flow Chart.

To assess reach, recruitment strategies are summarized in Table 1, with Facebook ads identified as the most frequently reported source of enrollment. The enrollment rate among those who accessed the study survey page was 65% (1349/2054; Figure 1). The retention rate at 1 month postassessment for participants randomized at preassessment was 42% (576/1345). Results are reported in compliance with the CONSORT-EHEALTH (Consolidated Standards of Reporting Trials of Electronic and Mobile Health Applications and Online Telehealth) checklist [41] (see Multimedia Appendix 1).

Descriptive statistics for the sample at preassessment are presented in Table 1. Preassessment levels of all study variables did not significantly differ based on randomization to the intervention and control groups (Table 1). Additionally, there were no statistically significant differences between participants who completed both the pre- and postassessments and those who only completed the preassessment (Table 2).

**Table 1:** Characteristics of the intervention and control groups at preassessment (N=1345).

Characteristics			Intervention group (n=949)		Active control group (n=396)		*P* value^a^
Age (years), mean (SD)^b^			47.22 (16.46)		47.11 (16.68)		.91
**Gender, n (%)**							.85
	Women	764 (80.5)		317 (80.1)			
	Men	164 (17.3)		70 (17.7)			
	Other	21 (2.2)		9 (2.3)			
**Primary outcomes, mean** **(** **SD)**							
	Well-being^c^	42.82 (21.80)		43.96 (21.77)		.38	
	Psychosocial flourishing^d^	39.04 (9.82)		38.43 (10.10)		.30	
	Positive psychological health^e^	17.71 (6.76)		17.27 (7.02)		.28	
**Recruitment strategy, n (%)**							.75
	Facebook group	120 (12.6)		56 (14.1)			
	Facebook ad	736 (77.6)		302 (76.3)			
	Referral from a friend	11 (1.2)		5 (1.3)			
	Not available	82 (8.6)		33 (8.3)			

^a^Assessed with independent sample *t* tests (ie, 2-tailed and unpaired) or *χ*^2^ analysis.

^b^n=933 for the intervention group and n=390 for the active control group.

^c^Measured using the 5-item World Health Organization Well-Being Scale.

^d^Measured using the Flourishing Scale.

^e^Measured using the Mental Health Continuum-Short Form.

**Table 2:** Comparison of participants who completed postassessment versus those who did not complete postassessment (N=1345).^a^

Characteristics		Completed postassessment (n=576)	No postassessment (n=769)	*P* value^b^
Age (years), mean (SD)		47.01 (16.25)	47.32 (16.73)	.74
**Gender, n (%)**				.35
	Female	467 (81.1)	614 (79.8)	
	Male	100 (17.4)	134 (17.4)	
	Other	9 (1.6)	21 (2.7)	
**Primary outcomes, mean (SD)**				
	Well-being^c^	43.29 (22.78)	43.06 (21.03)	.85
	Psychosocial flourishing^d^	39.02 (10.46)	38.73 (9.47)	.59
	Positive psychological health^e^	17.93 (6.98)	17.32 (6.72)	.10
**Recruitment source, n (%)**				.49
	Facebook group	77 (13.4)	99 (12.9)	
	Facebook ad	443 (76.9)	595 (77.4)	
	Referral from a friend	4 (0.7)	12 (1.6)	
	Not available	52 (9.0)	63 (8.2)	

^a^For age n=569 for those who completed postassessment and n=754 for those with no postassessment.

^b^Assessed with independent sample *t* tests or *χ*^2^ analysis.

^c^Measured using the 5-item World Health Organization Well-Being Scale.

^d^Measured using the Flourishing Scale.

^e^Measured using the Mental Health Continuum-Short Form.

### Primary Outcomes

Results for the within-group changes for both the intervention and control groups are presented in Table 3. Significant improvements were observed in the WHO-5 (intervention: Cohen *d*=0.26, *P*<.001; control: Cohen *d*=0.24, *P*<.001), FS (intervention: Cohen *d*=0.19, *P*<.001; control: Cohen *d*=0.18, *P*<.001), and PWB (intervention: Cohen *d*=0.17, *P*=.001; control: Cohen *d*=0.24, *P*<.001) scales when measuring changes from pre- to postassessment. Between-group effects were estimated using an analysis of changes approach, controlling for preassessment scores in the models (H1). Although both groups showed significant improvements at postassessment, no significant between-group differences were found in the changes for WHO-5, PWB, and FS from pre- to postassessment (Table 4).

Next, we examined whether the effectiveness of the intervention varied by gender or age after controlling for participation/engagement and user satisfaction (H2). The models included the main effects (gender or age and group) as well as the interaction effects between gender and group or age and group. We found no significant interaction effects of age or gender by group on the change scores of the primary outcomes (*P* values for interaction effects ranged from .46 to .94; full results are available upon request). This indicates that the results for the intervention and active control groups did not significantly differ based on age or gender.

In the next analysis (H3), we compared group differences in retention and participation/engagement. There was no significant difference in retention rates between the intervention and control groups, *χ*_1_^2^ (N=1345)=0.30, *P*=.57. Additionally, there was no statistically significant difference in self-reported participation/engagement at postassessment between the 2 groups (β=–.007, *P*=.92).

**Table 3:** Within-group effects for the intervention and active control groups with the intention-to-treat analysis (N=1345).^a^

Outcomes			Preassessment, mean (SD)		Postassessment, mean (SD)		Pre-post				
							Wald		*P* value		Cohen *d*
**Intervention group (n=949)**											
	Well-being^b^	42.82 (21.80)		48.74 (23.26)		25.23		<.001		0.26	
	Psychosocial flourishing^c^	39.04 (9.82)		40.98 (10.69)		13.18		<.001		0.19	
	Positive psychological health^d^	17.72 (6.76)		18.91 (7.16)		10.57		.001		0.17	
**Active control group (n=396)**											
	Well-being^b^	43.97 (21.75)		49.39 (23.62)		22.19		<.001		0.24	
	Psychosocial flourishing^c^	38.43 (10.10)		40.20 (9.89)		19.42		<.001		0.18	
	Positive psychological health^d^	17.28 (7.01)		18.94 (6.69)		29.45		<.001		0.24	

^a^Results indicate that in the intent-to-treat analyses, accounting for missing data at post, there is a significant improvement in well-being and flourishing for participants in the intervention group (Cohen *d*=0.17-0.26) and the active control group (Cohen *d*=0.18-0.24).

^b^Measured using the 5-item World Health Organization Well-Being Scale.

^c^Measured using the Flourishing Scale.

^d^Measured using the Mental Health Continuum-Short Form.

**Table 4:** Between-group effects comparing the intervention and active control groups with the intention-to-treat analysis (N=1345).^a^

Between-group effects	Mean post-pre change (SD), intervention group	Mean post-pre change (SD), active control group	β	SE	Estimated (SE)	*P* value
Well-being^b^	5.93 (16.00)	5.58 (17.64)	.004	0.041	0.078 (0.787)	.92
Psychosocial flourishing^c^	1.95 (6.94)	1.78 (6.23)	.019	0.038	0.147 (0.297)	.62
Positive psychological health^d^	1.15 (5.15)	1.53 (4.84)	–.022	0.038	–0.132 (0.227)	.56

^a^Based on the estimated means with the intent-to-treat analyses, accounting for missing data at post, there are no significant differences between the intervention groups in effects on primary outcomes. Group is coded –1 for control and 1 for intervention, positive β values indicate more change in the intervention group compared with the control group. There is a trend that the intervention group shows more improvements for all measures over time but not for flourishing between pre- and postassessment; however, all differences were not statistically significant.

^b^Measured using the 5-item World Health Organization Well-Being Scale.

^c^Measured using the Flourishing Scale.

^d^Measured using the Mental Health Continuum-Short Form.

### Intervention Group Analyses

**Figure 2:**
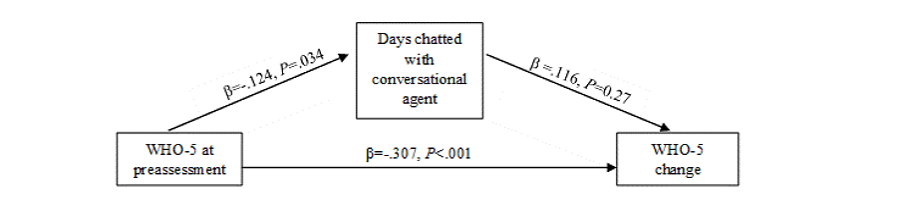
Days chatted with the conversational agent and associations with change in well-being (N=949). WHO-5 change indicates change in well-being. WHO-5: 5-item World Health Organization Well-Being Scale.

Descriptive statistics for the conversational agent interaction variables are reported in Table 5. Regarding H4, we examined whether user satisfaction, participation/engagement, and the intervention’s conversational agent measures of engagement were associated with greater improvements in well-being as measured by the WHO-5, FS, and PWB scales. Regression analyses were conducted separately for each predictor of outcomes in the intervention group (Table 6). User satisfaction and participation/engagement at postassessment were significantly associated with greater improvements in all primary outcomes (*P*=.04 to <.001). Participants with more unfinished modules (modules started but not completed) showed less improvement in positive psychological health. Conversely, participants who engaged with the conversational agent on more days experienced significantly greater changes in well-being (*P*<.04). The results indicate that lower well-being at preassessment (WHO-5) was associated with more days of active messaging within the intervention group, which, in turn, was linked to larger improvements in well-being over time (Figure 2). This indicates that the level of participation and engagement played a crucial role in driving change.

To examine the association between user satisfaction ratings and engagement in terms of messages with the conversational agent in the intervention group (H5), we conducted multilevel analyses that included elapsed time since enrollment, covering the first 30 days, in a 2-level model. Engagement was found to be negatively associated with time, indicating that there was greater engagement earlier in the 30-day period (n=435, β=–08, SE=0.03, *t*=–2.60, *P*=.009). Among the participants who provided user satisfaction ratings, the average number of ratings was 3.36 (SD 1.15) over the 30 days (n=211). The smaller sample size is attributed to some users not accessing the conversational agent. The module rating measure of user satisfaction significantly predicted the number of days messages were sent over the 30 days (n=211, β=.14, SE 0.03, *t*=4.31, *P*<.001). The association between average user satisfaction ratings and average participation/engagement across the 30 days approached statistical significance (*r*=0.13, *P*=.06).

**Table 5:** Descriptive statistics of conversational agent interaction data (implementation).^a^

Variables	Mean (SD)	Range
Days chatted with the bot^b^	4.26 (4.76)	1-29
Modules started^c^	3.68 (5.05)	0-32
Modules completed^d^	2.78 (4.69)	0-32
Modules started not completed^e^	0.90 (1.11)	0-10
Total messages^f^	51.09 (101.77)	0-740
Module rating of user satisfaction^g^	3.21 (1.00)	1-5

^a^Participants who engaged with the bot within the first 30 days: N=375, except for “days chatted with the bot” (n=339) and “module rating of user satisfaction” (n=211).

^b^Number of days the user messaged the conversational agent within the first 30 days.

^c^Number of modules started (finished and unfinished modules) within the first 30 days.

^d^Number of modules completed (finished modules) within the first 30 days.

^e^Number of modules started but not completed (unfinished modules) within the first 30 days.

^f^Count of messages sent by the user to the conversational agent within the first 30 days.

^g^Average rating of modules within the first 30 days.

**Table 6:** Implementation variables predicting well-being change outcomes from conversational agent and survey responses (n=949).

Variables		Well-being^a^		Psychosocial flourishing^b^		Positive psychological health^c^	
		β	*P* value	β	*P* value	β	*P* value
**Postassessment measures**							
	User satisfaction^d^	.296	<.001^e^	.222	.002^f^	.206	.001^f^
	Participation/engagement^g^	.236	<.001^e^	.153	.04^h^	.146	.04^h^
**Intervention conversational agent measures**							
	Total messages^i^	.068	.15	–.006	.89	–.020	.66
	Days chatted with the bot^j^	.109	.04^h^	–.014	.78	–.024	.62
	Modules completed^k^	.064	.18	–.001	.98	–.019	.67
	Modules started^l^	.066	.18	–.007	.88	–.038	.41
	Modules started not completed^m^	.032	.56	–.033	.50	–.116	.07
	Module rating of user satisfaction^n^	.152	.13	.185	.08	.134	.13

^a^Measured using the 5-item World Health Organization Well-Being Scale.

^b^Measured using the Flourishing Scale.

^c^Measured using the Mental Health Continuum-Short Form.

^d^User satisfaction scale with 4 items on satisfaction, usefulness, relevance, and helpfulness.

^e^*P*<.001.

^f^*P*<.01.

^g^Reported frequency spent using the program or control condition at the postassessment (2 items).

^h^*P*<.05.

^i^Count of messages sent by the user to the conversational agent within the first 30 days.

^j^Number of days the user messaged the conversational agent within the first 30 days (days active).

^k^Number of modules completed within the first 30 days.

^l^Number of modules started within the first 30 days.

^m^Number of modules started but not completed (unfinished modules) within the first 30 days.

^n^Average rating of modules within the first 30 days.

## Discussion

### Principal Findings

The primary aim of the study was to evaluate the effectiveness of a newly developed conversational agent–based self-care program (intervention group) compared with existing evidence-based wellness resources (active control group) on the primary outcomes of well-being (WHO-5), psychosocial flourishing (FS), and PWB scales. As anticipated, both groups showed significant improvements from pre- to postassessment in well-being (WHO-5 and PWB) and flourishing (FS). This indicates that with intention-to-treat analyses, which accounted for missing data at postassessment and adjusted for preassessment levels in the analytical model, significant improvements were observed in both well-being measures and psychosocial flourishing for participants in both the intervention and active control groups, with small effect sizes. However, contrary to our hypothesis, there were no significant differences between the groups when comparing changes from pre- to postassessment on the outcomes, as both groups demonstrated improvements. Taken together, participants in both groups—the conversational agent self-care program (intervention) and the evidence-based wellness resources (active control)—showed improvements at postassessment. This suggests that both interventions may offer public health benefits for prevention, especially given their no-cost format. However, future studies should compare these interventions with other active intervention conditions and evaluate how they perform against no-intervention conditions.

This is in line with previous studies. A randomized controlled trial in Poland tested an agent-guided cognitive behavioral therapy (Fido) for reducing symptoms of anxiety and depression, comparing it with an active control group that used self-help books. The authors aimed to replicate findings from earlier studies, such as those involving Woebot. Their results showed that both groups (intervention and control) demonstrated reductions in anxiety and depressive symptoms postintervention [42]. By contrast, the Woebot study found a reduction in depressive symptoms only in the intervention group, not in the control group [21]. Similarly, a systematic review and meta-analysis found that conversational agent–based interventions yield promising results in reducing symptoms of depression and psychological distress, but no significant improvement in well-being was observed [20]. One possible explanation for the inconsistent findings regarding well-being, as suggested by Li and colleagues [20], is that measures of psychological distress tend to be more sensitive to change than measures of well-being. This implies that achieving significant improvements in well-being may require sustained, long-term engagement, or that different measures may be needed to detect small changes that could still hold public health significance.

No significant differences were found between groups regarding implementation outcomes, such as retention and engagement/participation, nor by gender or age. While the overall results did not indicate differences between groups, analyses within the intervention group revealed that user satisfaction and engagement with *Zenny* were related to changes in well-being outcomes. These findings underscore the importance of examining both implementation and effectiveness outcomes together and highlight the need to focus on enhancing user satisfaction and engagement in digital interventions to improve well-being.

Thus, while conversational agent–based interventions show promise for promoting mental health, most studies demonstrating improvements in mental health outcomes either lacked a control group or used inactive control groups. Furthermore, when comparing conversational agent–based interventions with an active control group, many studies either did not achieve significant between-group effects [17] or found significant results only for depressive symptoms and psychological distress, with no notable improvements in other outcomes, such as well-being [20]. Additionally, most interventions that included an active control group showed significant short-term effects but lacked long-term efficacy [18]. Research also indicates that agent-based interventions are more effective in clinical and subclinical settings than in nonclinical contexts [20]. This aligns with earlier findings that psychological interventions tend to be more effective for individuals with mental or physical health issues compared with those in community samples [43]. Therefore, further research is needed that includes active control groups and investigates the long-term efficacy of conversational agent–based interventions across various mental health outcomes, including well-being, using a universal prevention approach.

### Strengths and Limitations

The use of WhatsApp posed a barrier to participation in the intervention group but not in the active control group, which may have affected the results by reducing exposure to the intervention. Consequently, implementation data (eg, module ratings, total messages) were available only for the intervention group. A potential solution would be to conduct the next study using WhatsApp for both groups to control for this difference or to implement the conversational agents on other platforms, such as a web browser.

Our study featured a newly developed conversational agent, which should be taken into account when interpreting the findings. While much of the qualitative feedback on the agent was positive—participants found it helpful, easy to access, and effective in identifying areas for personal change—some participants in the intervention group also provided negative feedback. This included issues such as reminders not working and occasionally slow responses. Additionally, recent research contradicts earlier findings [44] that advocated for the use of engagement reminders in digital interventions. A recent meta-analysis [18] revealed that not including automatic reminders in conversational agent–based interventions had a stronger positive effect on reducing depressive symptoms. This suggests that frequent reminders may hinder user interest rather than motivate them. According to this meta-analysis, future studies should prioritize personalization and empathetic responses, as these factors are strongly linked to the effectiveness of conversational agent–based interventions.

Furthermore, the conversational agent was still under development during data collection (eg, bug fixes), indicating that the actual effect size of the intervention should be interpreted with caution. While we selected resources that provided similar evidence-based self-help content for common risk factors affecting well-being in the active control condition, we did not systematically collect process data to compare the intervention content. This presents a potential direction for future research. Future studies should focus on strategies to enhance engagement in the intervention conditions and evaluate engagement with different types of content. Additionally, while participants were not aware of the content in the other condition, it was not possible to blind them during the intervention. Lastly, men and participants under the age of 35 years are significantly underrepresented in the sample. Furthermore, we did not assess other demographic variables, such as education, marital status, or employment status, to keep the participant survey brief. As a result, we cannot determine whether the findings are generalizable based on these characteristics.

Despite these limitations, the study has several strengths. It reports the initial results of a new conversational agent with a relatively large sample size, utilizing a randomized controlled trial design and including both men and women across a wide age range. The effect sizes observed for both the conversational agent and the web-based resource condition, although small, may still hold public health relevance due to their use of freely available interventions.

### Conclusion and Implications

This study compared 2 digital self-help programs aimed at promoting subjective well-being. Many self-help programs are available with limited evidence of their effectiveness, and this study helps address that gap by conducting a randomized trial of 2 such programs. Both the conversational agent and the evidence-based web resources demonstrated small improvements in well-being, but did not significantly differ in effectiveness. Additionally, the study highlighted the importance of engagement for achieving change and emphasized the need for future research focused on enhancing reach, engagement, and effectiveness in freely accessible self-help programs.

## Appendix

Multimedia Appendix 1 CONSORT-eHEALTH checklist (V 1.6.1).

## Abbreviations

CONSORT-EHEALTH: Consolidated Standards of Reporting Trials of Electronic and Mobile Health Applications and Online Telehealth
FS: Flourishing Scale
MHC-SF: Mental Health Continuum-Short Form
PWB: Psychological Well-Being
WHO-5: 5-item World Health Organization Well-Being Scale
